# Supplementary figures and images for: Mononuclear cell dynamics in M. tuberculosis infection provide opportunities for therapeutic intervention
Source: PLoS Pathog. 2018 Oct 26;14(10):e1007154. doi: 10.1371/journal.ppat.1007154 (PMC6221360; doi:10.1371/journal.ppat.1007154)

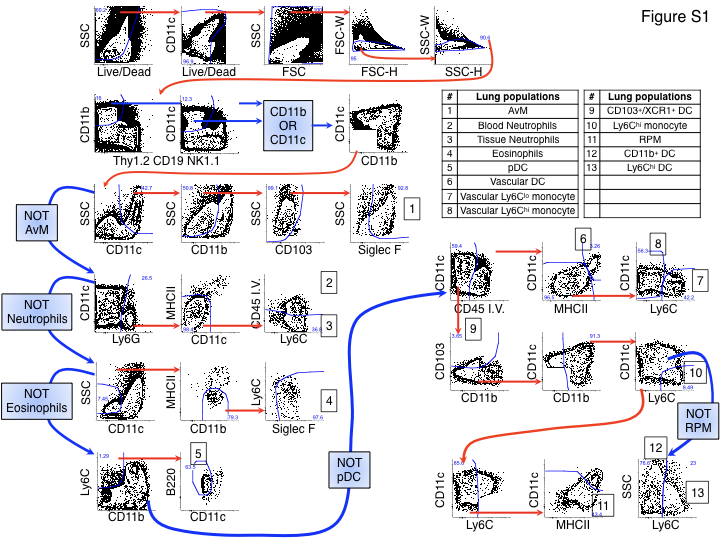

Supplement: S1 Fig — Representative gating strategy to identify adoptively transferred mononuclear cell subsets within the lungs of M. tuberculosis-infected mice. Samples are concatenated data from 5 mice from a single time point of a single time point, 8 weeks after infection with M. tuberculosis. Red gates are traditional sequential gates, while blue gates represent Boolean gating, where every cell not defined by a particular combination of markers of a cell population are then considered for further analysis. (TIF) [file ppat.1007154.s001.tif]

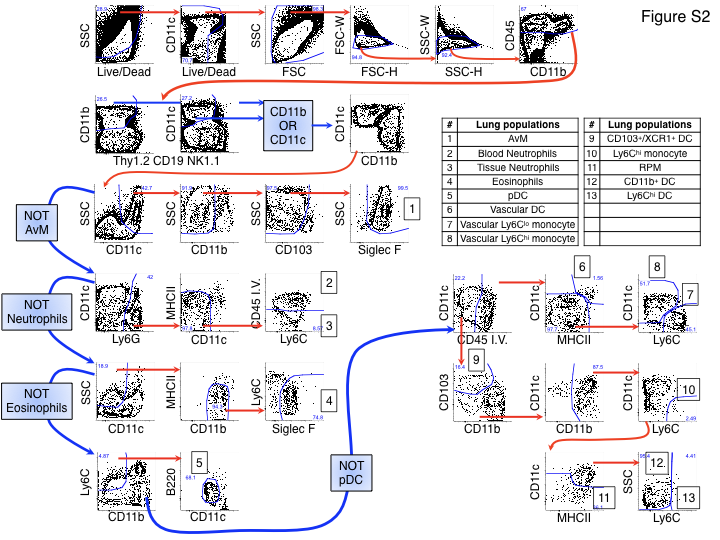

Supplement: S2 Fig — Representative gating strategy to identify vascular and parenchymal mononuclear cell subsets in the lungs of uninfected mice. Samples are concatenated data from 5 mice from a single time point. Red gates are traditional sequential gates, while blue gates represent Boolean gating. (TIF) [file ppat.1007154.s002.tif]

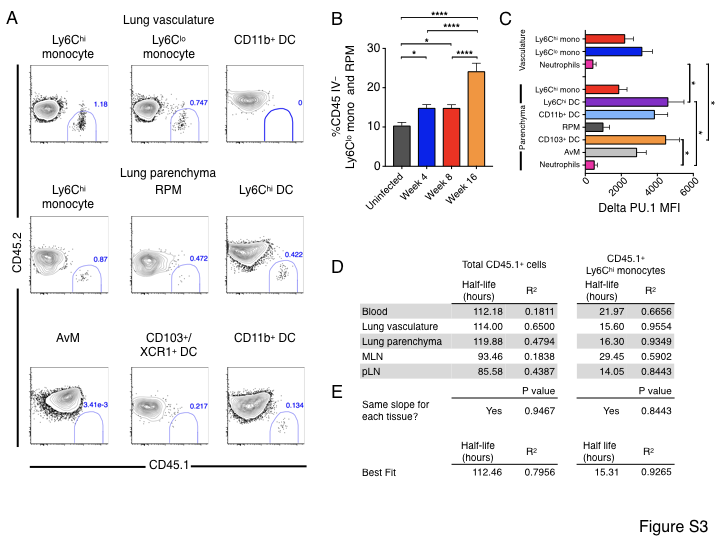

Supplement: S3 Fig — Bone marrow monocytes (CD45.1+) were depleted of stem cells and transferred to M. tuberculosis-infected mice (CD45.2+). A) Representative plots of CD45.1 donor-derived mononuclear cell subsets in the lungs of mice 40 hours after transfer to M. tuberculosis-infected mice. B) The proportion of Ly6CloCD11b+CD11cloMHCII− cells that are within the parenchyma increases as M. tuberculosis infection increases. Data are from 1–4 experiments per infection phase with 29–30 mice per week per experiment. C) Mean fluorescent intensity (MFI) of professional phagocyte populations in the lungs of mice infected for 8 weeks with M. tuberculosis. Data are calculated by subtracting the background MFI of fluorescence minus one (FMO) control samples from the PU.1 MFI and are presented as the mean ± SEM of pooled samples from 5 mice and 4 time points of a single experiment. D) Calculated half-life of donor total donor CD45.1 cells or Ly6Chi monocytes and R2 coefficient of determination. E) Comparison of fits for the slopes of the best-fit lines for total donor cells and donor Ly6Chi monocytes in each tissue. The null hypothesis is that all slopes are equal, and each equation is compared to a best-fit equation calculated from all data points. Data from D and E are from a single representative experiment of two experiments and are presented as means ± SEM of 5 mice per time point. (TIF) [file ppat.1007154.s003.tif]

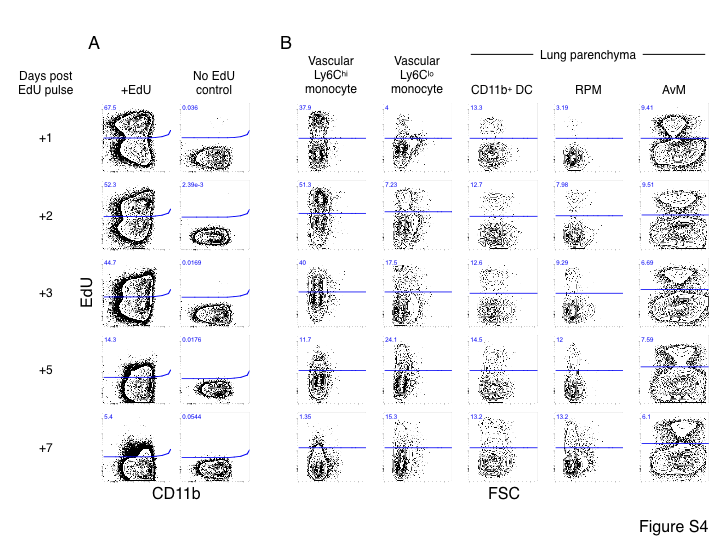

Supplement: S4 Fig — A) Representative gating of EdU staining in the bone marrow from EdU-injected mice and uninjected controls across multiple time points after EdU pulse. B) Representative gating of EdU staining in multiple mononuclear cell populations in the lungs of uninfected mice. Samples are concatenated data from 5 mice per time point and EdU gates were set relative to EdU-unpulsed uninfected controls. (TIF) [file ppat.1007154.s004.tif]

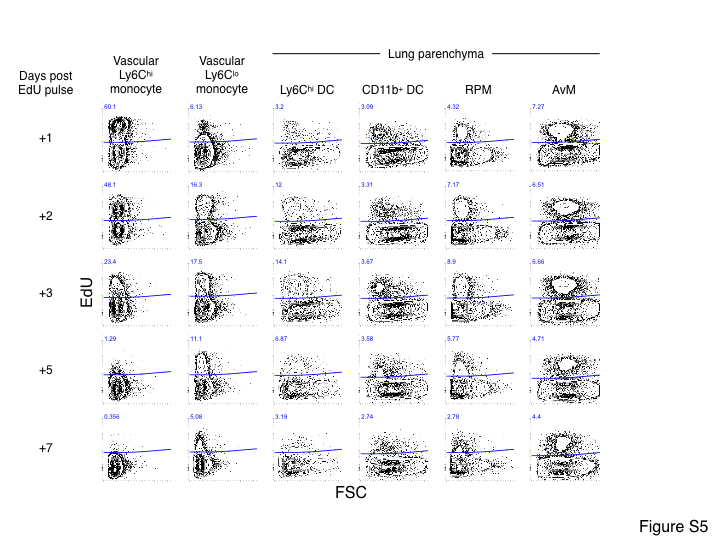

Supplement: S5 Fig — Representative gating of EdU staining in multiple lung mononuclear cell subsets at multiple time points following EdU injection, 8 weeks after infection with M. tuberculosis. Samples are concatenated data from 5 mice per time point and EdU gates were set relative to EdU-unpulsed M. tuberculosis-infected controls. (TIF) [file ppat.1007154.s005.tif]

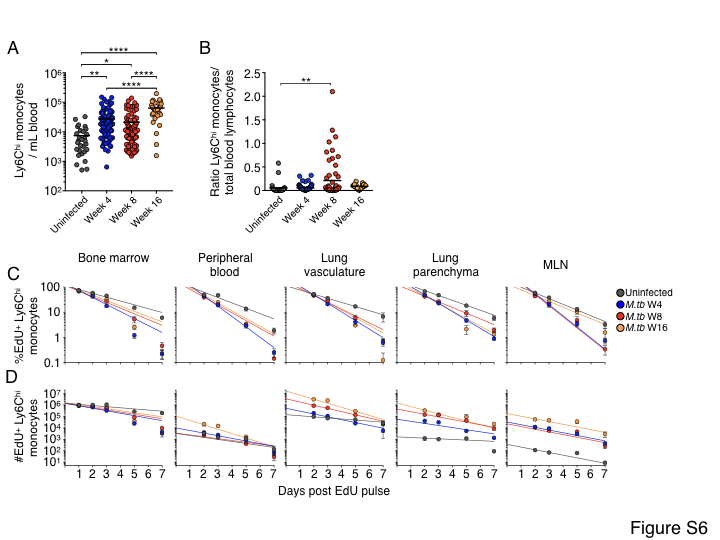

Supplement: S6 Fig — Monocytosis in the blood of M. tuberculosis-infected mice is matched by an increase in the rate of Ly6Chi monocyte turnover. Peripheral blood monocytosis during chronic M. tuberculosis infection relative to naïve mice, as determined by A) total Ly6Chi monocytes per mL of blood, and B) ratio of Ly6Chi monocytes to total lymphocytes in the blood. Data are presented as blood monocytes in individual mice from 1–4 experiments per infection phase with 29–30 mice per week, per experiment. C) Frequency of EdU+ staining in Ly6Chi monocytes in multiple tissues of uninfected and M. tuberculosis-infected mice and the exponential best-fit line. Statistics are ordinary one-way ANOVA with Sidak’s multiple comparisons tests. *p<0.05, **p<0.01, ***p<0.001, ****p<0.0001. D) Total numbers of EdU+ Ly6Chi monocytes in multiple tissues of uninfected and M. tuberculosis-infected mice and the exponential best-fit line. Data are means from 1–4 experiments per infection phase with 4–5 mice per time point per experiment. (TIF) [file ppat.1007154.s006.tif]

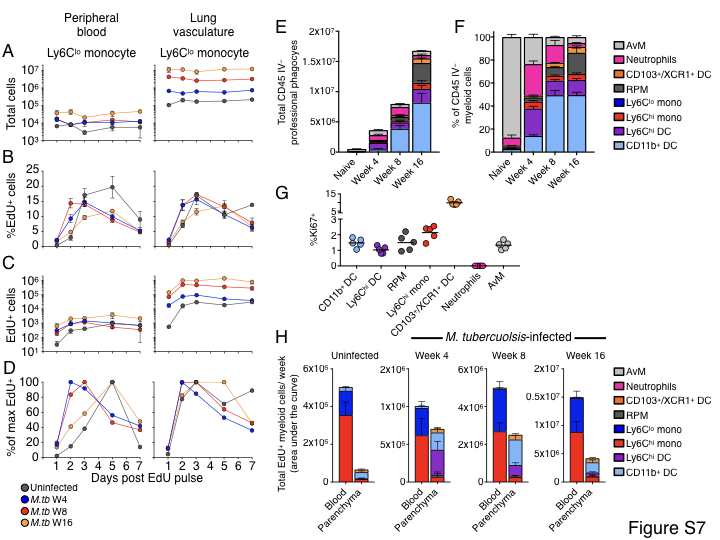

Supplement: S7 Fig — M. tuberculosis-infected mice were injected with EdU and its incorporation by mononuclear cell subsets was evaluated by flow cytometry at multiple time points. A) Total numbers, frequency EdU+ B) and total numbers C) of EdU+ peripheral blood and lung vasculature Ly6Clo monocytes in uninfected mice or mice pulsed with EdU 4 weeks, 8 weeks and 16 weeks after infection with M. tuberculosis. Data are means and SEM from 1–4 experiments per infection phase with 4–5 mice per time point per experiment. D) Mean percent of maximum frequency-of-live cells for EdU+ Ly6Clo monocytes in the lung vasculature and circulation of uninfected mice or mice pulsed with EdU 4 weeks, 8 weeks and 16 weeks after infection with M. tuberculosis. E) Total numbers and relative frequency F) of CD45 IV− professional phagocytes in the lung parenchyma of M. tuberculosis-infected mice at multiple phases of infection. G) Frequency of Ki67+ staining on multiple professional phagocytes from individual mice in the lung parenchyma 4 weeks after M. tuberculosis infection. Data are presented as individual mice from a single experiment. H) Total area under the curve of vascular and parenchymal EdU+ mononuclear cell subsets in the lungs of uninfected mice or M. tuberculosis-infected mice pulsed with EdU at weeks 4, 8 and 16 post infection. Data are means and SEM from 1–4 experiments per infection phase with 4–5 mice per time point per experiment. (TIF) [file ppat.1007154.s007.tif]

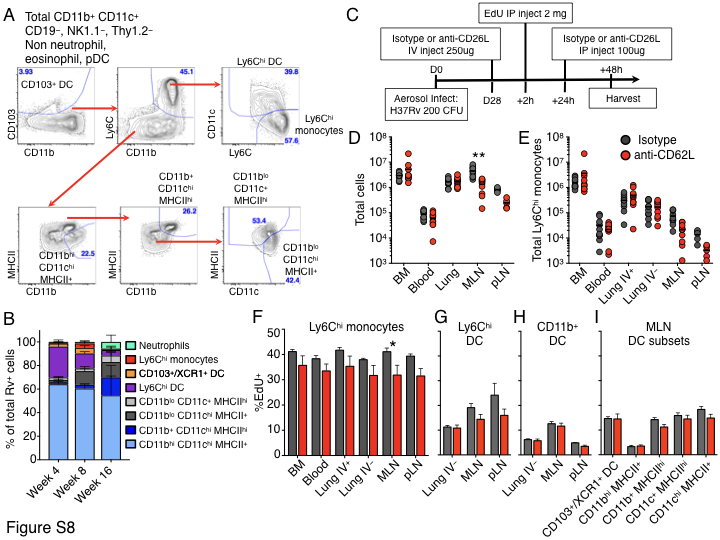

Supplement: S8 Fig — M. tuberculosis-infected mice were injected with EdU and its incorporation by mononuclear cell subsets in the MLN was evaluated by flow cytometry at multiple time points. A) Representative gating hierarchy for multiple mononuclear cell subsets within the MLN of mice infected with M. tuberculosis. B) Relative frequency of professional phagocytes containing mCherry-expressing M. tuberculosis in the MLN of M. tuberculosis-infected mice at multiple phases of infection. Data are presented as means ± SEM of 1–3 experiments with 5 mice per time point. C) Schematic showing the experimental design to block monocyte trafficking to the LN through the HEV. D) Total numbers of live cells and total numbers of Ly6Chi monocytes (E) in multiple tissues and in the blood in mice day 30 post-infection and following two days of isotype or anti-CD62L antibody treatment. F) Frequency of EdU incorporation by Ly6Chi monocytes in multiple tissues and the blood two days following EdU pulse of week 4 M. tuberculosis-infected mice. G) Frequency of EdU incorporation in Ly6Chi DC and total CD11b+ DC (H) in the lung parenchyma, MLN and pLN. I) Frequency of EdU incorporation in multiple mononuclear cell subsets in the MLN. Data from D-I are presented as means ± SEM from 2 experiments with 4–5 mice per time point per experiment. Statistics are 2-way ANOVA with Sidak’s multiple comparisons tests. *p<0.05. (TIF) [file ppat.1007154.s008.tif]

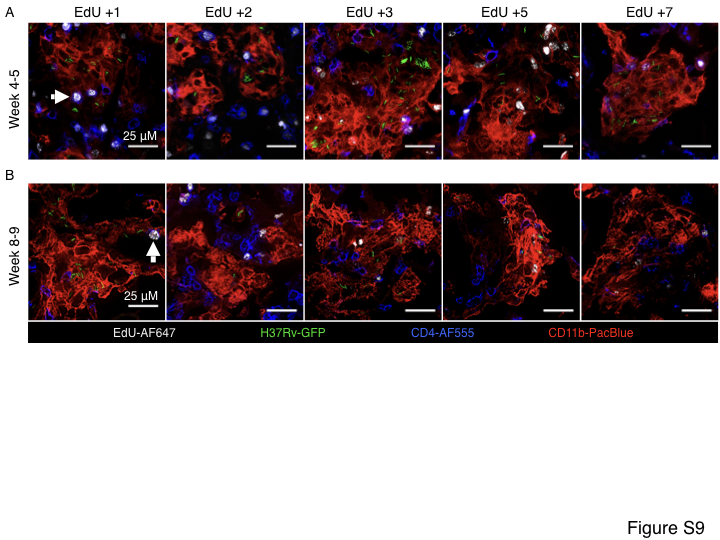

Supplement: S9 Fig — Mice infected with fluorescent protein-expressing M. tuberculosis were injected with EdU and its incorporation by dividing mononuclear cells evaluated by fluorescence microscopy at multiple times. Representative immunofluorescent staining of lung granulomas at multiple multiple time points following EdU pulse, 4 (A) or 8 weeks (B) after infection with GFP-expressing M. tuberculosis. Images are representative of 1–2 mice per time point. (TIF) [file ppat.1007154.s009.tif]

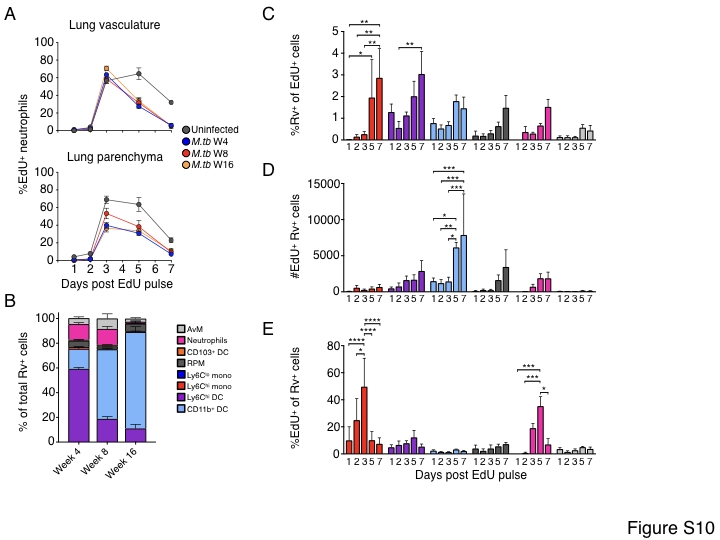

Supplement: S10 Fig — Mice infected with fluorescent protein-expressing M. tuberculosis were injected with EdU and its incorporation by dividing myeloid cells evaluated by flow cytometry at multiple time points. A) Frequency of EdU+ neutrophils in the lung vasculature and parenchyma of uninfected mice or mice pulsed with EdU 4 weeks, 8 weeks and 16 weeks after infection with M. tuberculosis. Data are presented as means and SEM from 1–4 experiments per infection phase with 4–5 mice per time point per experiment. B) Composition of total lung cells infected with mCherry-expressing M. tuberculosis at multiple phases of infection. Data are presented as means and SEM from 1–4 experiments with 5 mice per time point. C) Frequency of Rv+ cells within EdU+ mononuclear cells in the lung parenchyma of mice pulsed with EdU 16 weeks after infection with M. tuberculosis. D) Total numbers of Rv+ EdU+ mononuclear cells in the lung parenchyma of mice pulsed with EdU 16 weeks after infection with M. tuberculosis. E) Flow cytometric analysis of the frequency of Rv-mCherry-infected professional phagocytes that were EdU+ at multiple time points following EdU pulse 16 weeks post infection. Data are presented as means ± SEM of 4–5 mice per time point from a single infection for each phase. Statistics are 2-way ANOVA with Sidak’s multiple comparisons tests. *p<0.05, **p<0.01, ***p<0.001, ****p<0.0001. (TIF) [file ppat.1007154.s010.tif]

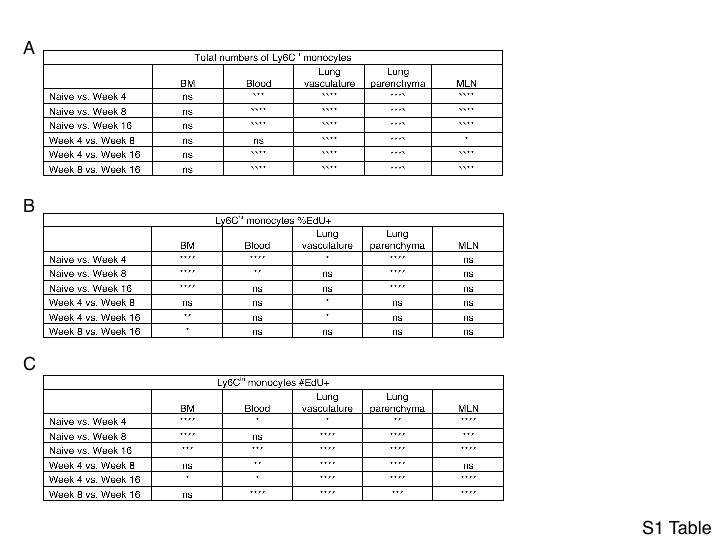

Supplement: S1 Table — Statistical analysis of A) total number of Ly6Chi monocytes, B) %EdU staining and C) total number of EdU+ Ly6Chi monocytes in uninfected and M. tuberculosis-infected mice. Statistics are two-way ANOVA comparing each phase. p<0.05, **p<0.01, ***p<0.001, ****p<0.0001, ns = not significant. (TIF) [file ppat.1007154.s011.tif]

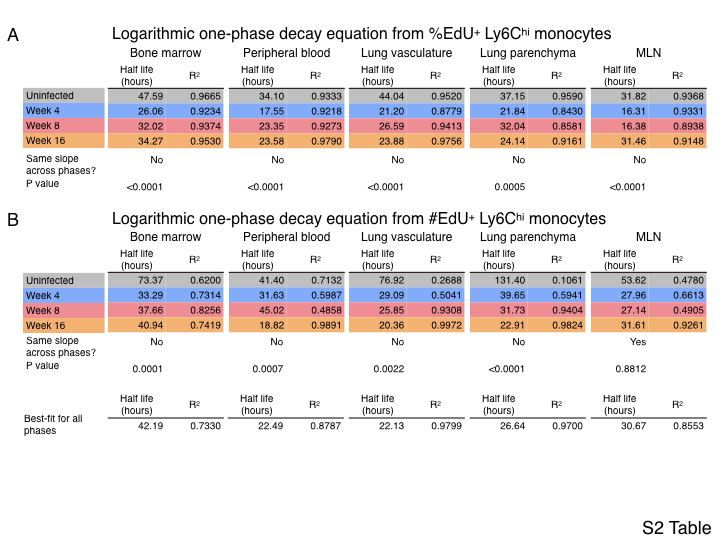

Supplement: S2 Table — M. tuberculosis-infected mice were injected with EdU and its incorporation by dividing monocytes was evaluated by flow cytometry at multiple time points. Exponential one-phase decay calculations were performed for Ly6Chi monocytes during each infection phase and in each tissue. A) Tabulated results of half-lives calculated from exponential best-fit lines of %EdU+ Ly6Chi monocytes and the R2 coefficient of determination. Comparison of fits for the slopes of the best-fit lines of %EdU+ Ly6Chi monocytes for each organ from infected or naïve mice. Null hypothesis is that the slope is the same for all each tissue, independent of infection phase. B) Tabulated results of half-lives calculated from exponential best-fit lines of the total number of EdU+ Ly6Chi monocytes and the R2 coefficient of determination. Comparison of fits for the slopes of the best-fit lines of total number of EdU+ Ly6Chi monocytes for each organ from infected or naïve mice. Null hypothesis is that the slope is the same for all each tissue, independent of infection phase. Calculated best-fit of all infection phases for each organ and the R2 coefficient of determination. (TIF) [file ppat.1007154.s012.tif]

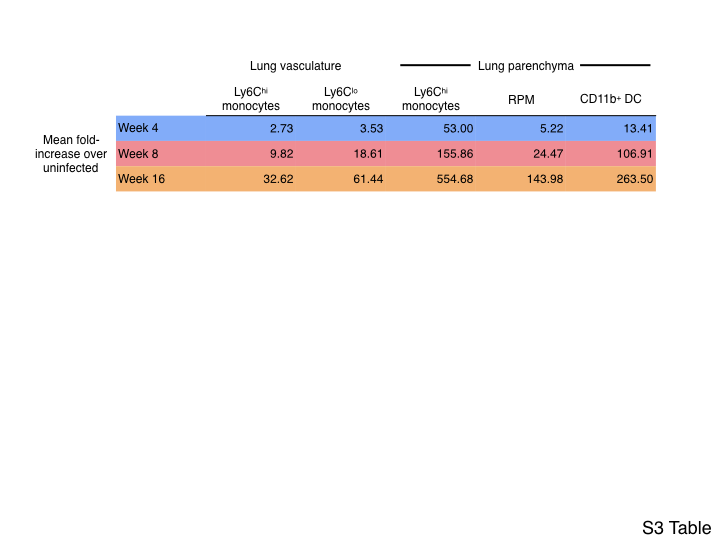

Supplement: S3 Table — Mean fold-increase of lung mononuclear cell populations at multiple phases of M. tuberculosis infection, relative to uninfected mice. Data are means from 1–4 experiments per infection phase with 4–5 mice per time point per experiment. (TIF) [file ppat.1007154.s013.tif]

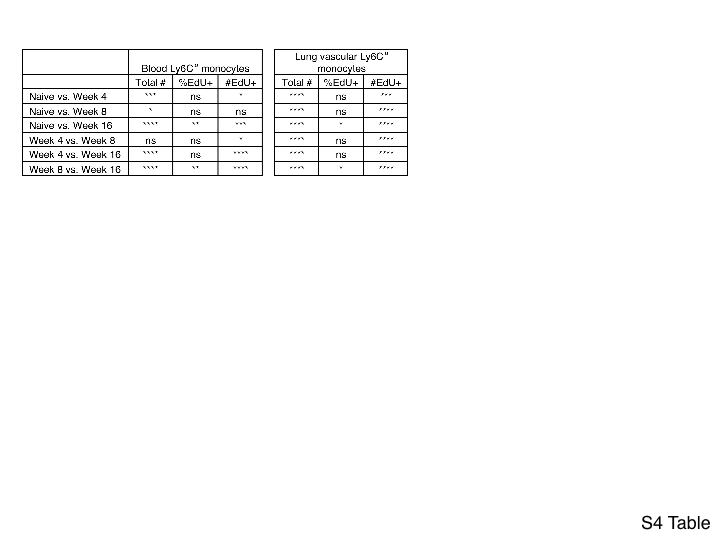

Supplement: S4 Table — Statistical analysis of total number, %EdU staining and total number of EdU+ Ly6Clo monocytes or RPM in the blood or lung vasculature, respectively, of uninfected and M. tuberculosis-infected mice. Statistics are two-way ANOVA comparing each phase. p<0.05, **p<0.01, ***p<0.001, ****p<0.0001, ns = not significant. (TIF) [file ppat.1007154.s014.tif]

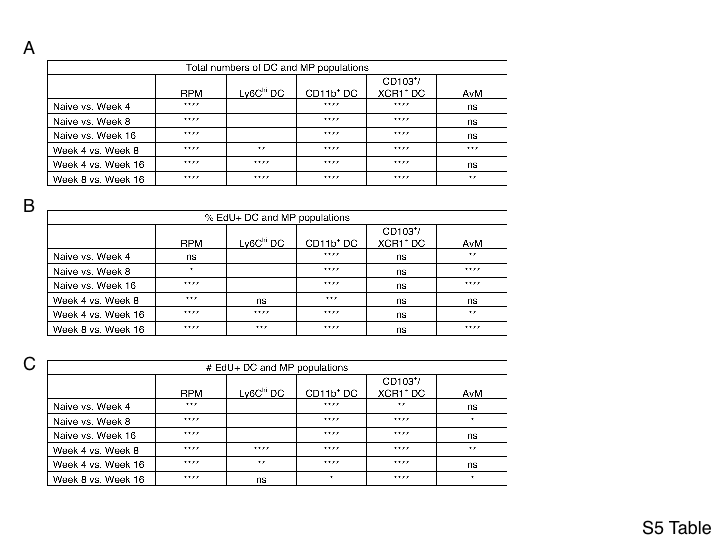

Supplement: S5 Table — Statistical analysis of A) total number, B) %EdU staining and C) total number of EdU+ MP or DC populations in uninfected and M. tuberculosis-infected mice. Statistics are two-way ANOVA comparing each phase. p<0.05, **p<0.01, ***p<0.001, ****p<0.0001, ns = not significant. (TIF) [file ppat.1007154.s015.tif]
