# Supplementary material for: Mononuclear cell dynamics in M. tuberculosis infection provide opportunities for therapeutic intervention
Source: PLoS Pathog. 2018 Oct 26;14(10):e1007154. doi: 10.1371/journal.ppat.1007154 (PMC6221360; doi:10.1371/journal.ppat.1007154)
Supplement: S6 Table — Statistical analysis of A) total number, B) %EdU staining and C) total number of EdU+ mononuclear cell populations in uninfected and M. tuberculosis-infected mice. Statistics are two-way ANOVA comparing each phase. p<0.05, **p<0.01, ***p<0.001, ****p<0.0001, ns = not significant. (DOCX) [file ppat.1007154.s016.docx]

A

| Total numbers of MLN mononuclear cell subsets | | | | | |
| --- | --- | --- | --- | --- | --- |
|  | CD103^+^/  XCR1^+^ DC | CD11b^hi^  CD11c^hi^  MHCII^+^ | CD11b^+^  CD11c^hi^  MHCII^hi^ | CD11b^lo^  CD11c^+^  MHCII^hi^ | CD11b^lo^  CD11c^hi^  MHCII^+^ |
| Naive vs. Week 4 | **** | **** | **** | **** | **** |
| Naive vs. Week 8 | **** | **** | **** | **** | **** |
| Naive vs. Week 16 | **** | **** | ** | **** | **** |
| Week 4 vs. Week 8 | ns | ns | ** | ns | **** |
| Week 4 vs. Week 16 | **** | **** | **** | * | **** |
| Week 8 vs. Week 16 | **** | ** | ** | *** | **** |

B

| %EdU+ MLN mononuclear cell subsets | | | | | |
| --- | --- | --- | --- | --- | --- |
|  | CD103^+^/  XCR1^+^ DC | CD11b^hi^  CD11c^hi^  MHCII^+^ | CD11b^+^  CD11c^hi^  MHCII^hi^ | CD11b^lo^  CD11c^+^  MHCII^hi^ | CD11b^lo^  CD11c^hi^  MHCII^+^ |
| Naive vs. Week 4 | ns | ns | ns | ns | * |
| Naive vs. Week 8 | ** | ns | ** | ns | ns |
| Naive vs. Week 16 | ns | ** | ns | *** | ** |
| Week 4 vs. Week 8 | *** | ns | ** | * | ** |
| Week 4 vs. Week 16 | ns | ns | ns | ns | ns |
| Week 8 vs. Week 16 | **** | * | **** | *** | ** |

C

| %EdU+ MLN mononuclear cell subsets | | | | | |
| --- | --- | --- | --- | --- | --- |
|  | CD103^+^/  XCR1^+^ DC | CD11b^hi^  CD11c^hi^  MHCII^+^ | CD11b^+^  CD11c^hi^  MHCII^hi^ | CD11b^lo^  CD11c^+^  MHCII^hi^ | CD11b^lo^  CD11c^hi^  MHCII^+^ |
| Naive vs. Week 4 | *** | **** | **** | ** | **** |
| Naive vs. Week 8 | *** | *** | **** | **** | **** |
| Naive vs. Week 16 | **** | **** | **** | **** | **** |
| Week 4 vs. Week 8 | * | ns | ns | * | ns |
| Week 4 vs. Week 16 | **** | **** | * | * | **** |
| Week 8 vs. Week 16 | **** | *** | ns | **** | **** |
